# Adhesion of *Neisseria meningitidis* to Dermal Vessels Leads to Local Vascular Damage and *Purpura* in a Humanized Mouse Model

**DOI:** 10.1371/journal.ppat.1003139

**Published:** 2013-01-24

**Authors:** Keira Melican, Paula Michea Veloso, Tiffany Martin, Patrick Bruneval, Guillaume Duménil

**Affiliations:** 1 INSERM, U970, Paris Cardiovascular Research Center, Paris, France; 2 Université Paris Descartes, Faculté de Médecine Paris Descartes, Paris, France; 3 AP-HP, Hôpital Européen Georges Pompidou, Paris, France; University of Oxford, United Kingdom

## Abstract

Septic shock caused by *Neisseria meningitidis* is typically rapidly evolving and often fatal despite antibiotic therapy. Further understanding of the mechanisms underlying the disease is necessary to reduce fatality rates. *Postmortem* samples from the characteristic *purpuric* rashes of the infection show bacterial aggregates in close association with microvessel endothelium but the species specificity of *N. meningitidis* has previously hindered the development of an *in vivo* model to study the role of adhesion on disease progression. Here we introduced human dermal microvessels into SCID/Beige mice by xenografting human skin. Bacteria injected intravenously exclusively associated with the human vessel endothelium in the skin graft. Infection was accompanied by a potent inflammatory response with the secretion of human inflammatory cytokines and recruitment of inflammatory cells. Importantly, infection also led to local vascular damage with hemostasis, thrombosis, vascular leakage and finally *purpura* in the grafted skin, replicating the clinical presentation for the first time in an animal model. The adhesive properties of the type IV pili of *N. meningitidis* were found to be the main mediator of association with the dermal microvessels *in vivo*. Bacterial mutants with altered type IV pili function also did not trigger inflammation or lead to vascular damage. This work demonstrates that local type IV pili mediated adhesion of *N. meningitidis* to the vascular wall, as opposed to circulating bacteria, determines vascular dysfunction in meningococcemia.

## Author Summary

Certain bacterial pathogens access the bloodstream during infection and this is associated with extremely severe conditions such as septic shock. A central feature of these infections is the rapid alteration of blood vessel function with deregulated inflammation, coagulation and loss of vessel integrity. Studying the mechanisms of infection of *Neisseria meningitis in vivo* for the first time, we show that the ability of this bacterium to adhere to and proliferate in the blood vessel, a process we refer to as vascular colonization, is a prerequisite to the alteration of vascular function. Previously, circulating bacteria were thought to be responsible. We identified the bacterial factors involved in this process by showing that it is largely dependent on type IV pili, long filamentous appendages that allow bacteria to stick to the vessel walls. To study infection by *N. meningitidis*, a highly human specific pathogen, we introduced human vessels into mice by grafting human skin onto immunodeficient mice. This work thus also introduces a new animal model of infection that reproduces the cardinal features of meningococcal infections. Such a model will be useful to the scientific community to explore the mechanisms of disease, and test new treatment or preventive strategies.

## Introduction

Fulminant meningococcal septic shock is the most lethal outcome of infection with the human-specific bacterium *Neisseria meningitidis* with a case fatality rate of 16–52% [1]. Despite the apparent virulence of the bacteria, between 5 and 30 % of the human population carry *N. meningitidis* in their throat asymptomatically [1]. Pathology is initiated when the pathogen accesses the bloodstream, and can lead to distinctive disease progressions, meningitis (37–49%), septic shock (10–18%) or a combination of the two (7–12%) [1]. Meningococcal septic shock, either alone or in addition to meningitis, is typically rapidly evolving and responsible for 90% of fatal cases [2]. Despite antibiotic treatment, severe *sequelae* and death rates remain high for meningococcal septic shock, and a better understanding of the mechanisms of the infection is required.

Clinical studies have provided a detailed description of the late stages of the disease. *Post mortem* histological studies have shown bacterial aggregates inside the lumen of blood vessels in several organs including skin, liver, brain, and kidney [3], [4], [5], [6]. A characteristic site of infection is the skin. Dermal lesions, including petechial and *purpura* rashes, are considered one of the cardinal features of meningococcal septic shock, occurring in 28–78% of cases [1], [7]. Bacteria can be isolated from the skin of 86% of patients with meningococcal sepsis by needle aspiration [8]. Close association of bacterial aggregates with endothelial cells suggests that bacteria are adhering along the vessel wall although this remains to be demonstrated [3], [6].

The presence of bacteria inside microvessels is associated with a potent inflammatory response and vascular damage. Infection triggers the secretion of high levels of inflammatory cytokines including IL-6, IL-8 and TNFα in patient serum [9], [10], [11]. A cellular infiltrate primarily consisting of monocytes and polymorphonuclear neutrophils (PMN) is observed in infected areas [3], [4], [12] and bacteria are frequently found inside these phagocytic cells [4], [6]. Widespread thrombosis of the small vessels is associated with alterations in blood flow, congestion of red blood cells and subsequently dilation and engorgement of the vessels. Endothelial damage results in vascular leakage, and the development of *purpura* [1], [5], [12], [13]. Despite the strong association of meningococcal disease and *purpura* as well as the serious prognosis associated with it, the bacterial factors involved and the sequence of events leading to the histological observations remain poorly understood. How the bacterial aggregates form inside the vessels is still unknown. It may be that bacteria associate as pre-formed aggregates that get lodged in the microvasculature, possibly proliferate after reduction in circulation following coagulation, or it may be an active adhesion process.

One of the major obstacles in the study of meningococcal septic shock and meningitis has been the absence of an experimental model, primarily due to the strong human specificity of *N. meningitidis*. Adhesion to endothelial cells is a factor likely to contribute to the difficulty in developing animal models that reproduce the human disease. *N. meningitidis* adhesion to human cells in culture is several orders of magnitude more efficient than to mouse cells *in vitro* [14]. A variety of bacterial adhesins have been described *in vitro* including type IV pili (tfp), Opa proteins, OpC, TspA or NadA but their relative importance for adhesion *in vivo* is unknown [15], [16], [17], [18].

The objective of this work was to investigate the impact of bacterial adhesion to the vessel on vascular dysfunction during septic shock and *purpura in vivo*. We report the development of a humanized mouse model for *N. meningitidis* based on the previously described xenografting of human skin onto SCID/Beige mice [19]. In this model the grafted human dermal microvasculature anastomoses with the mouse circulation thus introducing functional human microvessels in these animals. We show that infection of this humanized mouse model results in extensive bacterial adhesion, which leads to vascular dysfunction and reproduces the cutaneous lesions found in acute meningococcemia.

## Results

### Grafting of human skin onto SCID/Beige mice introduces functional human microcirculation

Our first objective was to develop an experimental model for meningococcal infection, which included bacterial adhesion. Our strategy was to introduce human dermal vessels into a mouse model. Human skin, 200 µm thick, including the corneum, epidermis and the papillary layer, containing an abundance of capillaries, was grafted onto SCID/Beige mice [19].

**Figure 1:**
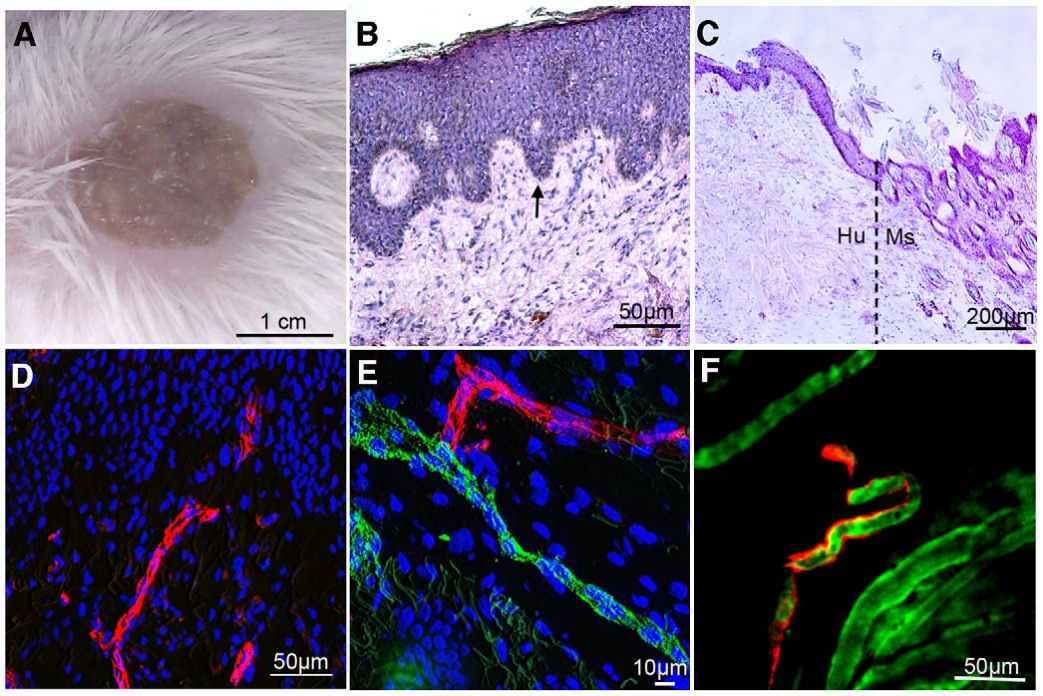
Human skin graft. (A) 21 days post graft of human skin onto SCID/Beige mouse. (B) Histology of grafted human skin showing dermal/epidermal border (arrow); (H&E). (C) Graft interface (dotted line) between human (Hu) and mouse (Ms) skin (H&E). (D) Human endothelium in the grafted skin labeled with *Ulex europaeus agglutinin* lectin (UEA, red). Cell nuclei are labeled with DAPI (blue). (E) Junction between human (UEA lectin, red) and mouse (msCD31, green) vessels at the graft border. (F) Frame from Movie S1. Intravital microscopy showing perfusion of human vessels labeled with UEA lectin (red). Blood plasma is labeled with 150 kDa FITC-dextran (green). Black silhouettes within the flow are red blood cells.

**Figure 2:**
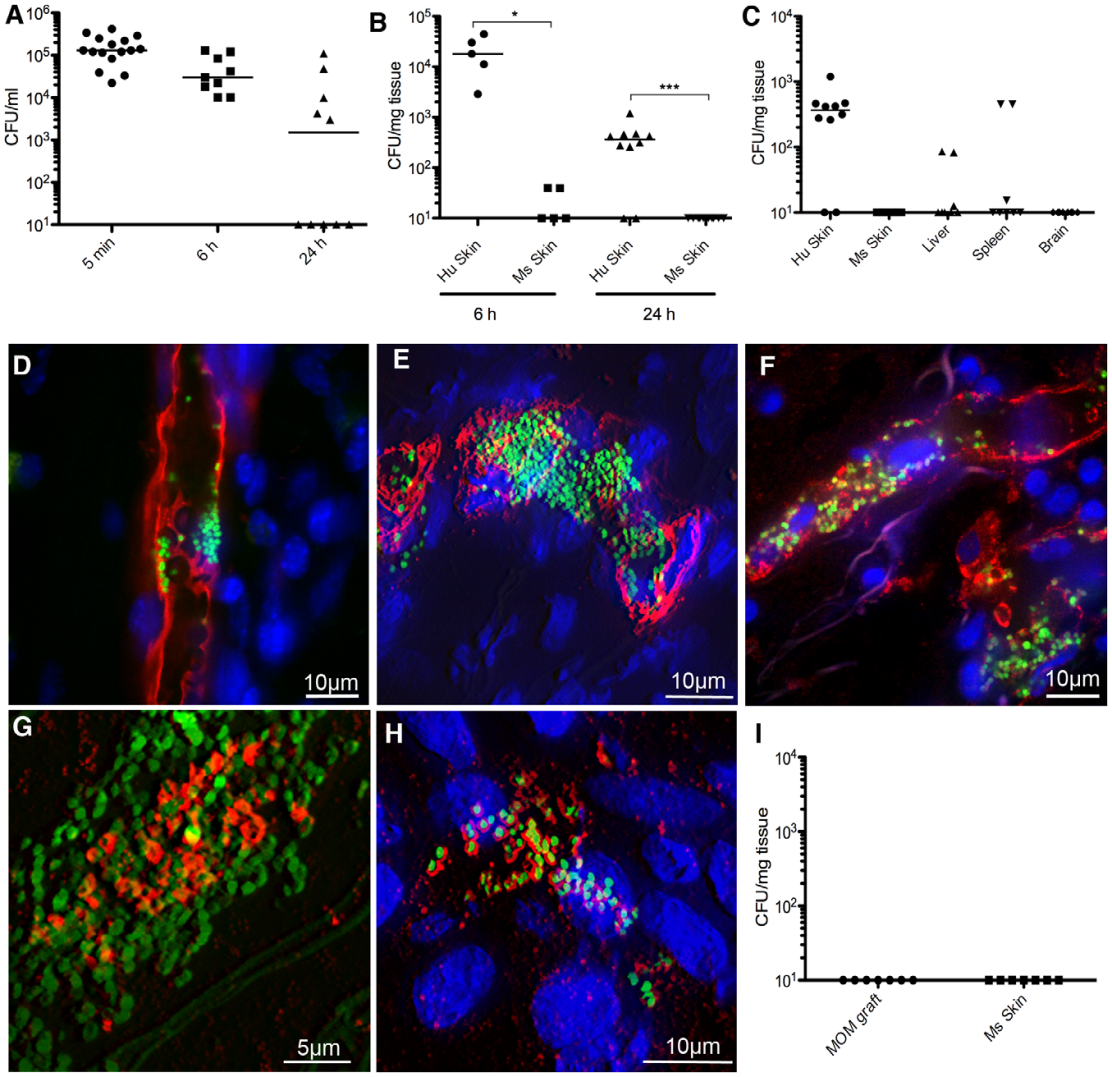
Bacterial association with vessels. (A) Bacterial colony forming unit (CFU) counts from the blood of mice grafted with human skin and infected with *N. meningitidis*. These counts represent the number of bacteria circulating in the blood at the given time-point. (B) Bacterial CFU counts from skin biopsies taken from either grafted human skin (Hu Skin) or contralateral mouse skin (Ms Skin). (C) Bacterial CFU counts in biopsies taken from organs of mice grafted with human skin. (D) Confocal microscopy showing *N. meningitidis* (green) associated with human vessels (UEA lectin, red) in the skin, 2 h post infection. (E) 3D reconstruction showing bacteria (green) in a human vessel (UEA lectin, red) in the skin, 6 h post infection (F) Bacteria (green) in a human vessel (HuCD31, red), 24 h post infection. (G) Bacteria (green) expressing tfp (red) 24 h post infection. (H) Bacteria (green) expressing capsule (red), 24 h post infection. (I) CFU counts from skin biopsies of mice grafted with mouse skin (MOM graft, mouse on mouse), and contralateral mouse skin (Ms), after 24 h of infection with *N. meningitidis*.

Three to four weeks post graft (Fig. 1A), the human skin displayed classical morphology with a clearly detectable epidermis and dermis and no evident inflammation (Fig. 1B). The graft border between the human graft and mouse skin was identified by reduced epidermal thickness and presence of hair follicles in the mouse skin (dotted line, Fig. 1C). The human origin of the microvessels, and retention of human endothelial cells following grafting was confirmed by staining with the lectin *Ulex europaeus agglutinin* (UEA), a marker of human endothelial cells (Fig. 1D), as well as a monoclonal antibody specific for human CD31 (PECAM-1) (Fig. 2F). At the interface of the grafts, junctions between human and mouse vessels could be identified (Fig. 1E). Circulation of blood in the human vessels was demonstrated by intravital imaging. Introduction of fluorescently labeled UEA lectin and 150 kDa FITC-dextran in the circulation of the grafted animals allowed the identification of the human vessels and blood flow respectively. Red blood cells were visible in the human vessels as black silhouettes in the fluorescent plasma (Fig. 1F and Movie S1). This shows that grafting of human skin resulted in perfused, functional human vessels in the SCID/Beige mouse.

### *Neisseria meningitidis* associate with vessels in the grafted human skin

In patients presenting with fatal meningococcemia, the bacterial load, as determined by qRT-PCR, has a median value of approximately 10^5^ colony forming units (CFU)/ml [20]. To mimic this scenario, a total of 10^6^ CFU bacteria per mouse were introduced intravenously into 15 grafted mice leading to an average of 1.5×10^5^ CFU bacteria/ml effectively circulating within 5 min of injection (Fig. 2A). One aspect of the human specificity of *N. meningitidis* is iron uptake, necessary for bacterial growth [21], [22]. In line with previous findings [23], [24] intraperitoneal injection of human transferrin prior to infection increased bacterial loads at 6 h and therefore all experiments presented were performed with the addition of human transferrin (Fig. S1E). In 5 mice the infection was allowed to proceed for 6 h and in the other 10 mice for 24 h. These 15 mice represent data pooled from 4 separate experiments performed at different times using skin from 7 different donors. Bacterial loads in the blood averaged 4.8×10^4^ CFU/ml at 6 h and 2.4×10^4^ CFU/ml at 24 h (Fig. 2A). At 24 h 5/10 mice had no detectable bacteria in their circulation.

Bacterial counts were then determined from the grafted human skin and other organs. Bacterial counts were consistently high in the grafted human skin averaging 2.1×10^4^ CFU/mg of tissue after 6 h of infection, and 4.4×10^2^ CFU/mg after 24 h (Fig. 2B, Hu skin). In the same mice, contralateral mouse skin contained virtually no detectable bacteria (Fig. 2B, Ms skin). As a control of the grafting procedure, skin from C57/BL6 mice was grafted in place of the human skin on SCID/Beige mice. No bacteria were found in the grafted mouse tissue (Fig. 2I, MOM for ‘mouse on mouse’ graft). This data is pooled from 7 mice from 3 separate experiments. This data showed that *N. meningitidis* associated exclusively, and in significant numbers, to the microvessels in the grafted human skin. It is noteworthy that 3 of the 5 mice with no detectable bacteria in their blood at 24 h had substantial bacterial counts in the human skin graft. In general no bacteria were found in other organs after 24 h of infection except in 2 mice, with higher than average blood counts that showed bacteria in their liver and spleen (Fig. 2C).

Expression of green fluorescent protein (GFP) facilitated visualization of *N. meningitidis* in live animals and on tissue sections. Intravital imaging allowed for the observation of the initial steps of bacterial association with the vessel endothelium. At 30 min post infection individual bacteria and small aggregates were observed seemingly adhering to human blood vessels labeled with UEA lectin (Movie S2). Both live and fixed tissue analysis confirmed that bacteria were exclusively found in vessels staining positive for human endothelium (Fig. 2D–F). Bacteria were observed in the lumen of human vessels, either lining the endothelium or forming aggregates typically between 20 and 150 bacteria (Fig. 2D). Occasionally, bacteria filled the entire vessel (Fig. 2E). Bacterial aggregates were embedded in a heterogeneous but locally dense meshwork of type IV pili (Fig. 2G) and also expressed a polysaccharidic capsule forming a thick ring around individual bacteria (Fig. 2H). These data show that introduction of human vessels promoted the association of *N. meningitidis* with microvessels.

### hIL-6 and hIL-8 are secreted by infected human endothelium

**Figure 3:**
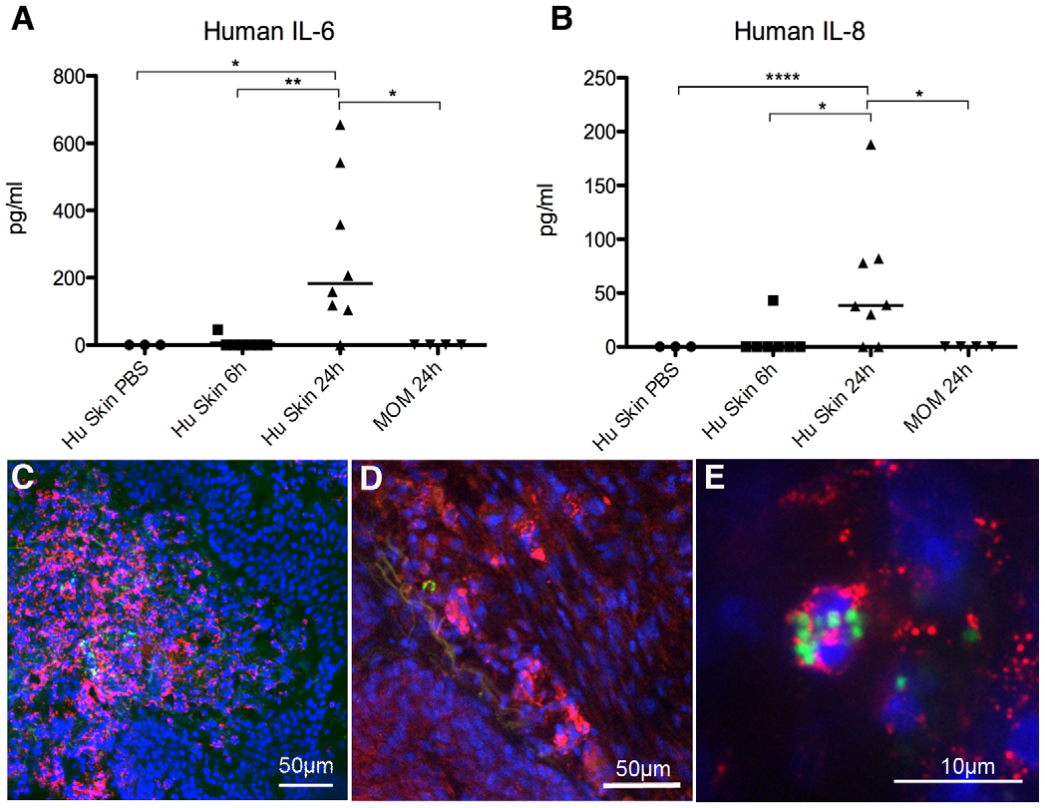
Inflammatory signaling following infection. (A) Human IL-6 concentrations in serum of mice grafted with human skin and injected with either PBS or *N. meningitidis*. MOM, control mice grafted with mouse skin and infected for 24 h. (B) Human IL-8 concentrations from serum of mice as in (A). (C) Neutrophil infiltration (Ly6G/Ly6C, red) at 24 h post infection, a few bacteria can be seen (green) in the inflamed area. Cell nuclei stained with DAPI (blue). (D) Neutrophil infiltration (red) at 6 h post infection. (E) *N. meningitidis* (green) phagocytosed by a neutrophil (red), 6 h post infection.

Abnormal regulation of inflammation, a ‘cytokine storm’, is an important feature of meningococcal septic shock [10], [11]. In this model we had the unique opportunity to differentiate the signaling occurring locally at the site of adhesion (human) from the circulating cells (mouse). Despite the small size of the human skin graft relative to the whole animal, expression of human IL-6 and IL-8 was detectable in the serum of human skin grafted animals, infected for 24 h (Fig. 3A–B, Hu skin 24 h). Human cytokine detection showed no cross reaction with purified mouse cytokines. No expression was seen in either of the control groups: (*i*) mice grafted with human skin and injected with PBS (Fig. 3A–B, PBS) or (*ii*) mice grafted with mouse skin and infected with *N. meningitidis* for 24 h (Fig. 3A–B, MOM). TNFα, IL-1α, IL-1β, IL-10, IFNγ, MIP1α, MIP1β, IFNα and GM-CSF were measured but not detected. As the circulating cells are of mouse origin it can be inferred that the human cytokines were produced by the endothelium. To confirm this, human endothelial cells (HUVEC) were exposed to *N. meningitidis in vitro* and they displayed the same cytokine profile with expression of only IL-6 (1200 pg/ml 24 h post infection) and IL-8 (1200 pg/ml, 24 h post infection) strengthening the implication of the endothelium as a key mediator of this response *in vivo*. These results point to a direct role of the endothelium in contributing to the IL-6 and IL-8 cytokine response seen in meningococcal patients.

**Figure 4:**
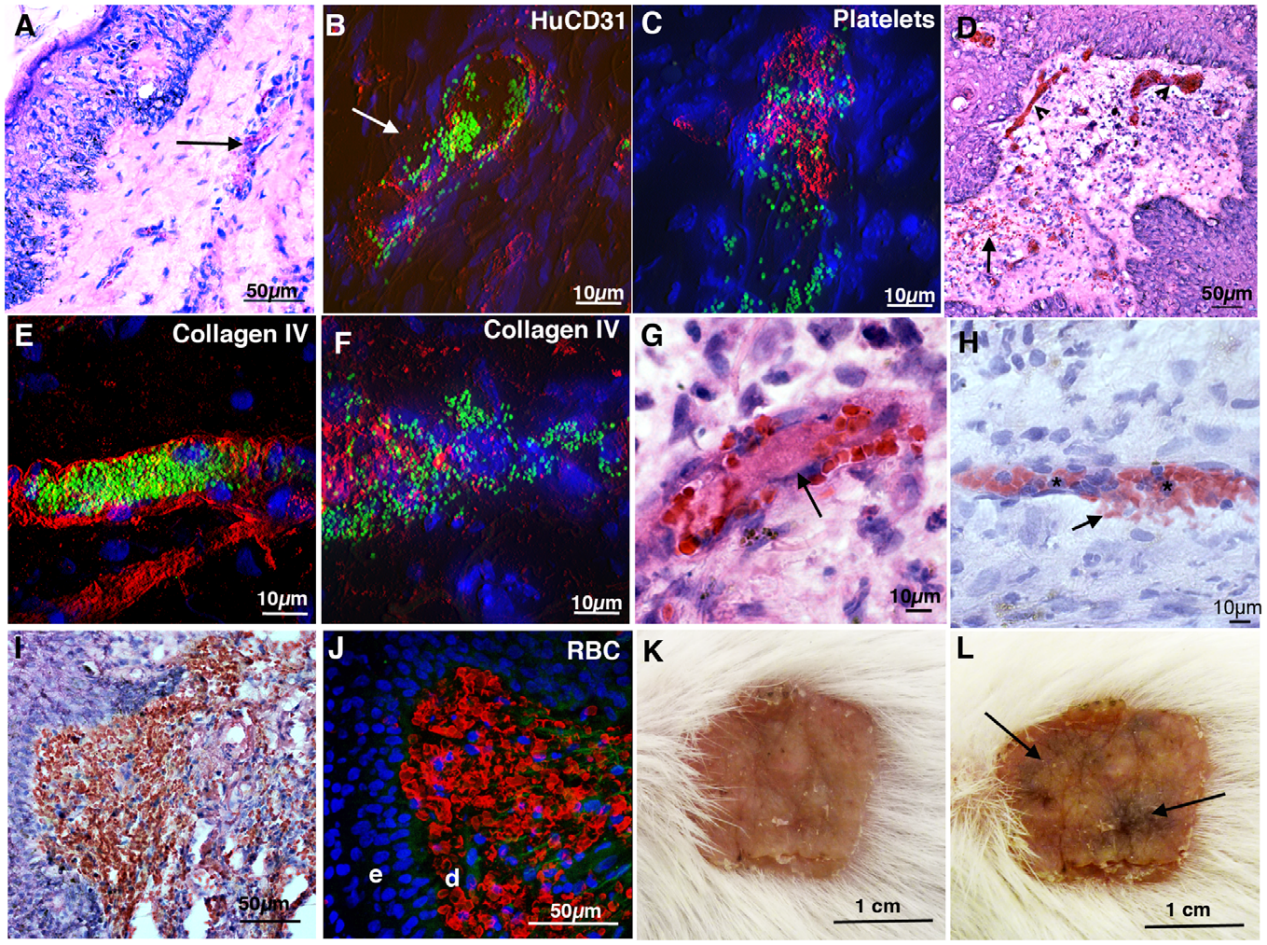
Tissue morphology following infection with *N. meningitidis*. (A) Tissue morphology 6 h post infection with *N. meningitidis* (H&E). Thrombosis and mild inflammation can be seen in a vessel (arrow). (B) Bacteria (green) at 6 h infection, human endothelium labeling (huCD31; red), indicates loss of continuous staining (arrow). Cell nuclei are labeled with DAPI (blue). (C) Platelet aggregation (red) in close proximity to bacteria (green), 6 h post infection. (D) Inflammation, vascular leak (arrow) and congestion (arrowheads) at 24 h post infection (H&E). (E) Bacteria (green) filling a vessel in close proximity to the basement membrane (Collagen IV, red). (F) Disruption of Collagen IV (red) in a heavily infected vessel at 24 h. (G) Thrombosed vessel (arrow) 24 h post infection (H&E). (H) Small vascular hemorrhage (arrow) in a congested vessel also showing neutrophil infiltration (*). (I) Extensive vascular leak, red/brown color shows large lake of RBCs at the dermal/epidermal border at 24 h (H&E). (J) RBC (red) leakage in the dermis (d). Leakage occurs at the epidermal (e) border. (K) Human skin graft prior to infection. (L) The same mouse as (K) 24 h post infection showing *purpura*, seen as dark areas (arrow).

Microscopy analysis revealed a massive recruitment of inflammatory cells to the skin at 24 h post infection (moderate in 6/10 and severe in 4/10; Fig. 3C, 4D,G, H and Table 1). Vessels were often filled with a combination of bacteria and infiltrating cells. Bacteria were commonly found phagocytosed by neutrophils (Fig. 3E). At 6 h post infection the recruitment was less pronounced (mild in 5/5; Fig. 3D, 4A and Table 1). Mice grafted with mouse skin and infected for 24 h had a very low level of inflammation (very mild 3/4, none in 1/4, Table 1). This data shows an increased infiltration of inflammatory cells as the infection progresses from 6 to 24 h.

**Table 1:** Histology scoring.

	Inflammation			Congestion			Thrombosis			Leakage		
	0/+	++	+++	0/+	++	+++	0/+	++	+++	0/+	++	+++
**PBS** 1	3	−	−	3	−	−	3	−	−	3	−	−
**MOM** 2	6	1	−	5	2	−	7	−	−	7	−	−
**WT 6 h**	5	−	−	3	2	−	4	1	−	4	1	−
**WT 24 h**	−	6	4	−	1	9	−	6	4	1	4	5
***pil*D 24 h**	5	−	−	4	1	−	4	1	−	5	−	−
***pil*C1 24 h**	1	4	−	2	3	−	5	−	−	5	−	−

0 = none detected.

+ = very mild/mild – occasional events detected, 1–2 per field of view.

++ = moderate – more than occasional, multiple events per field of view.

+++ = severe – dominant phenotype, most/all of field of view affected.

1 Mice grafted with human skin and injected with PBS 24h prior to sacrifice.

2 Mice grafted with mouse skin and infected with WT bacteria for 24 h.

### Association with the endothelium leads to cutaneous lesions

We next analyzed the impact of infection on vasculature integrity. At 6 h post infection the morphology of the tissue was generally well preserved although signs of mild vascular thrombosis and inflammation were apparent (Fig. 4A, arrow). Endothelial staining revealed occasional vessels with non-continuous staining (Fig. 4B, arrow), suggesting endothelial loss. Sloughed endothelial cells could be found in the lumen of larger vessels (Fig. S1A). Mild vascular congestion was seen in 2/5 mice with one mouse showing the first signs of vascular leakage (Table 1). Mild thrombosis in 3/5 mice was confirmed by staining of fibrin and platelet aggregation and was both in close proximity to and distal from bacterial colonies (Fig. 4C, and Fig. S1B–D).

At 24 h post infection tissue damage was extensive with widespread vascular damage (Fig. 4D). Staining of collagen IV, a major constituent of the basement membrane (BM), showed bacteria in close proximity to the BM, again suggesting loss of endothelium (Fig. 4E). In some vessels the integrity of the basement membrane appeared compromised (Fig. 4F). Vascular congestion and engorgement were consistently seen (moderate 1/10 and severe in 9/10) along with thrombosis (moderate in 6/10 and severe in 4/10; Fig. 4G, arrow). Vascular leakage was found in all infected mice (mild 1/10, moderate in 4/10, severe in 5/10; Table 1), varying from small perivascular hemorrhages (Fig. 4H, arrow) through to lakes of RBCs (Fig. 4I,J). Erythrocyte leakage was located primarily at the dermal/epidermal border (Fig. 4I,J). Importantly, 3/10 animals (30%) developed macroscopically identifiable non-blanching rashes reminiscent of clinical *purpura* (Fig. 4K, L). Control mice grafted with human skin and injected with PBS had typical non-infected morphology. Mice grafted with mouse skin and infected with bacteria had mild inflammation (moderate 1/7 and mild 7/7) and vascular congestion (moderate 2/7 and mild in 5/7) but no vascular leakage was detected (Table 1). Association of *N. meningitidis* with the human dermal vessels in this model led to vascular damage and cutaneous lesions replicating what is seen in clinical samples.

### Bacterial association with dermal vessels depends on adhesive type IV pili and leads to the development of cutaneous lesions

**Figure 5:**
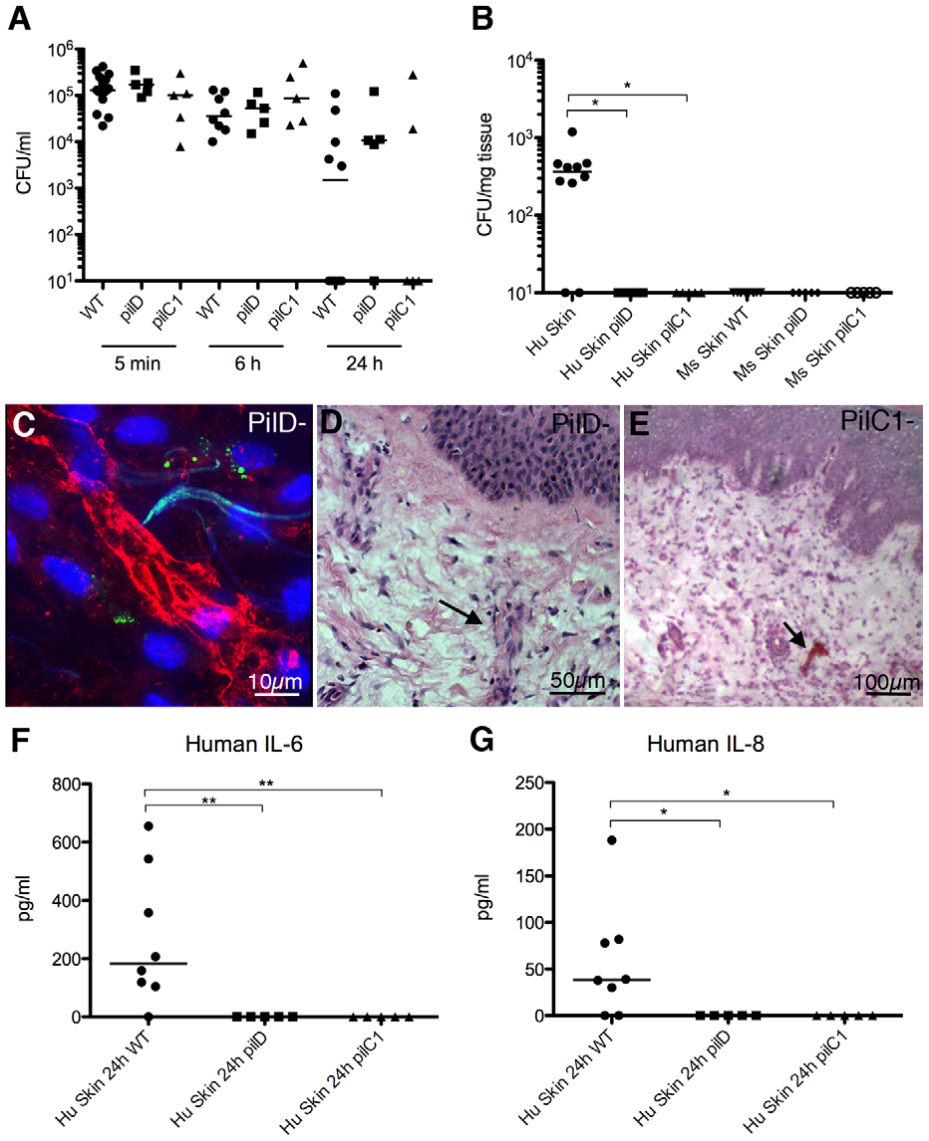
Infection with non-adherent mutants of *N. meningitidis*. (A) Bacterial CFU counts from the blood of mice infected with wild type *N. meningitidis* (WT), an isogenic non-piliated *pilD* mutant strain (*pilD*), and a piliated but non-adherent *pilC1* mutant strain (*pilC1*). (B) Bacterial counts of biopsies from grafted human skin and contralateral mouse skin of mice infected for 24 h as in (A). (C) 3D reconstruction of human vessel from a grafted mouse infected with the *pilD* mutant strain (UEA lectin, red). Small amounts of tissue auto-fluorescence and collagen can be seen in the green channel. (D) H&E staining of grafted human skin in a mouse infected with the *pilD* mutant strain. Arrow highlights very mild inflammation around a vessel. (E) H&E staining of grafted human skin in a mouse infected with the *pilC1* mutant strain. Arrow highlights signs of congestion in a vessel. (F) Cytokine analysis of human IL-6 in serum of mice grafted with human skin and infected with the WT or mutant strains. (G) Cytokine analysis of human IL-8 in the same mice as (F).

This experimental model of infection allowed for evaluation of the effect of specific *N. meningitidis* virulence factors in the process of vascular adhesion and cutaneous lesion development *in vivo*. Type IV pili (tfp) have been found to be a key mediator of adhesion *in vitro*. In addition, clinical strains are invariably piliated making tfp the prime candidate to mediate interaction with the vascular wall *in vivo*. To test the role of tfp we took advantage of two isogenic bacterial strains deficient in type IV pili function, *pilD* and *pilC1*. The *pilD* mutant is deficient in the biosynthesis of type IV pili and does not express any pili on its surface [25]. Infection of 5 human skin grafted mice with the *pilD* mutant strain did not reveal any significant difference in the circulating bacterial CFU counts in the blood as compared to the wild type (Fig. 5A, *pilD*, WT). No bacteria were however found associated with the human skin graft (Fig. 5B–C, Hu skin *pilD*). Data shown is from 2 separate infection experiments. This indicated that tfp are indeed important for bacterial association with the human vessels. To investigate specifically the adhesive properties of the tfp we used a *pilC1* mutant that displays pili on its surface but fails to mediate adhesion despite maintaining other type IV pili dependent properties such as bacterial aggregation [26]. Again circulating blood CFU counts of the *pilC1* mutant in 5 mice were not significantly different to wild type bacteria (Fig. 5A, *pilC1*). In the grafted human skin no *pilC1* mutant bacteria were detected (Fig. 5B, Hu skin *pilC1*). Data shown is from 2 separate infection experiments. This showed that it is the adhesive property of tfp that is essential for bacterial association with the human vascular endothelium *in vivo*.

Morphologically, tissues showed a low level of inflammation and neutrophil infiltration (mild in 5/5 *pilD* and 4/5 *pilC1*; Fig. 5D, arrow). Occasional vessels showed signs of congestion and thrombosis (none in 2/5 and mild in 3/5 *pilD*, mild 5/5 *pilC1*; Fig. 5E, arrow). No significant loss of vascular integrity or leakage was noted in either group (Table 1). Infection with neither the *pilD* or *pilC1* mutant strain resulted in any detectable expression of human IL-6 or IL-8 (Fig. 5F,G), suggesting tfp mediated adhesion is crucial for this endothelial signaling. This data shows that bacterial adhesion is mediated by tfp and that adhesion is essential for triggering the cascade of inflammation and vascular damage that results in cutaneous lesions *in vivo*.

## Discussion

Several attempts have been made to generate *in vivo* models of *Neisseria meningitidis* infection; each producing important advances [24], [27], [28], but lacking crucial aspects of the development of fulminant meningococcal septic shock. The human skin graft model described here reproduces several key features of acute infections caused by *N. meningitidis* [5], [13], [29]: (*i*) adhesion of bacteria to the microvasculature; (*ii*) induction of inflammation and (*iii*) vascular damage with coagulopathy and loss of vascular integrity. This model allows for studies aiming to understand the molecular mechanisms underlying the interaction of this bacterium with the microcirculation *in vivo* and the pathology of the cutaneous lesions which occur during meningococcemia. In addition, this model may prove to be an interesting new tool to evaluate new prevention strategies, which remains a key approach for the future due to the rapid evolution of meningococcemia. A large-scale effort is currently underway in industrial and academic research to design alternative vaccine antigens for serogroup B meningococcus [30], [31]. Evaluation of the efficiency of vaccines in experimental models is a critical preclinical step and remains a limitation in the development of meningococcal vaccines. The mouse background used in our xenograft model is unable to generate antibodies but serum generated in other mouse backgrounds against candidate antigens, passive immunization, could be tested in this model.

Although this model shows promise for future studies it cannot reproduce every aspect of the human infection. The human graft is a relatively small fraction of the organism and subsequently some systemic aspects of the human disease may be lacking. For instance, blood bacterial counts might be expected to increase after 24 h rather than decrease as we observed in some cases, although the dynamics of bacterial proliferation during the human disease is difficult to assess. It should also be noted that the crossing of the nasopharyngeal epithelium or the blood-brain barrier are not taken into account.

A major result of this study is that local bacterial adhesion to the endothelium is essential for triggering a cascade that results in cutaneous lesions *in vivo*. We also show that this adhesion *in vivo* is largely mediated by type IV pili. In contrast to type IV pili, which are expressed by all clinical strains, different strains express different sets of additional adhesins. The strain used in this study does not contain the *opc* gene and the *opa* genes are mostly in the OFF phase [32]. The possible participation of these adhesins will thus require further studies.

The pathogenic effects of meningococcemia, particularly on the skin have previously been attributed to circulating LPS [1], [33], [34]. In this model, non-adherent bacteria, despite circulating in the same numbers as the wild type strain and thus releasing similar amounts of LPS and other bacterial compounds, do not trigger cytokine secretion in the human tissue or lead to significant vascular damage. This highlights the importance of local adhesion events in the vasculature, a process we refer to as *vascular colonization* [35], in triggering the disease. This represents a change of paradigm from the idea that vascular damage is caused solely by large amounts of circulating bacteria and bacterial products in the blood. Once adherent to the endothelium, bacteria remain in tight aggregates and do not appear to seed the circulation in large quantities, as demonstrated by mice with no circulating bacteria despite high bacterial counts in the skin. Patients with meningitis frequently display no circulating bacteria in their blood [1] but based on our results large amounts of bacteria could still be bound to microvessels. These results point to the importance of designing new therapies targeting the interaction of the bacteria to the endothelium.

The infection of an average of 1 cm^2^/200 µm thick human skin graft into the mouse model was sufficient to enable the detection of human cytokines in the serum, emphasizing the intensity of the response following bacterial adhesion. The small size of the graft may however mean that other human cytokines that may be expected, such as TNFα, are not detected due to insufficient sensitivity. A previous report has shown a role for monocytic cells in the regulation of TNFα by endothelial cells [36] and the lack of human monocytes in this model may be another explanation to the lack of this cytokine. As the circulating cells are of mouse origin it can be inferred that the grafted human tissue produced the human cytokines. The combination of the *in vivo* data, the complimentary *in vitro* data and the fact that they are directly in contact with bacteria, suggests that the endothelium contributes to the secretion of these cytokines but in this study we cannot exclude the role other dermal cell types may play. While adhesion was the only factor definitively linked to increased cytokine production in this study, the intense inflammatory response seen in the tissue could also be related to the high local concentrations of bacterial factors such as LPS subsequent to adhesion. The alterations in blood flow or endothelial damage could also trigger the specific endothelial inflammatory signaling that is seemingly responsible for the recruitment of inflammatory cells. Further work will be needed to delineate the specific role of the inflammatory response in vascular damage.

Vascular colonization led to extensive vascular and tissue damage in the grafted human skin. It has been reported *in vitro* that large bacterial aggregates cause endothelial junctional openings on cells within 4 hours. This response depends on tfp and recruitment of junctional proteins and may be responsible for the vascular leak observed here [37], [38]. The animal model presented here provides information on the kinetics of meningococcal infection *in vivo* for the first time. At 6 h post infection vessel damage is relatively mild despite large numbers of bacteria whereas at 24 h, when damage is severe, bacterial quantity has not increased. This late onset of vascular leak is different to the junctional openings seen on isolated cells, suggesting that other mechanisms are involved *in vivo*. A major difference between the 6 and 24 h time-points is that the recruitment of inflammatory cells is much more pronounced at 24 h and a role for local inflammation in the alteration of vessel integrity and the development of the cutaneous lesion may be envisaged.

Macroscopically identifiable *purpura* or a petechial rash was seen in 30% (3/10) of the grafted mice despite the small size of the graft in this model, falling into the range of the 28–78% quoted for clinical cases [1], [2]. This result again points to the importance of the local events of infection as opposed to systemic infection as is frequently proposed. The *purpura* only developed where bacteria bind the vessel wall whereas circulating bacteria did not lead to such effects.

In conclusion, the experimental model depicted here faithfully reproduces key clinical features associated with meningococcal septic shock in the skin. By taking advantage of this new tool, we provide new insights to better understand the pathogenesis process by demonstrating the importance of local vascular colonization in triggering disease.

## Materials and Methods

### Ethics statement

All experimental procedures involving animals were conducted in accordance with guidelines established by the French and European regulations for the care and use of laboratory animals (Décrets 87–848, 2001–464, 2001–486 and 2001–131 and European Directive 2010/63/UE) and approved by the local ethical committee *Comité d'Ethique en matière d'Expérimentation Animale, Universite Paris Descartes*, Paris, France. No: CEEA34.GD.002.11. All surgery was performed under anesthesia, and all efforts were made to minimize suffering. For human skin, written informed consent was obtained and all procedures were performed according to French national guidelines and approved by the local ethical committee, *Comité d'Evaluation Ethique* de l'INSERM IRB 00003888 FWA 00005881, Paris, France Opinion: 11–048.

### Bacteria

*N. meningitidis* 8013 clone 12 is a serogroup C clinical isolate, expressing a class I SB pilin, Opa^−^, Opc^−^, PilC1^+^/PilC2^+^ [32]. *N. meningitidis* was grown on GCB agar plates (Difco, France) containing Kellogg's supplements and 100 µg/ml kanamycin, 50 µg spectinomycin or 5 µg/ml chloramphenicol as required at a humid 37°C, 5% CO_2_. Mutations in the *pilD* and *pilC1* genes were introduced into the *N. meningitidis* chromosome by natural transformation of chromosomal DNA, and selected with integrated antibiotic resistance cassette. The *pilD* mutation was extracted from a library of transposition mutants [39]. The *pilC1* mutant is described elsewhere [26]. *meningitidis* expressed the green fluorescent protein (GFP) under the control of the *pilE* promoter using the pGCC2 plasmid for chromosomal insertion [40]. A 300 base pair fragment of the *pilE* promoter was amplified from chromosomal DNA with the following primers: PrPilEF, 5′-AGTACTCCATGCCAATAGAGATACCCCACG-3′ introducing a *ScaI* site and PrPilER, 5′-TTAATTAAAATTGGAAAGGAAATGCCTCAAGC-3′ introducing a *PacI* site. The amplicon was cloned into the PCR2.1 Topo plasmid, sequence was checked, the insert restricted with *PacI* and *ScaI* and cloned into the pGCC2 vector restricted with the same enzymes generating the pGCC2PrPilE plasmid. The GFP ORF was amplified by PCR from the pAM239 [41] plasmid with the following primers: GFPF 5′-TTAATTAATTTAAGAAGGAGATATACATATGAGTAAAG-3′ introducing a *PacI* site and GFPR, 5′-GTCGACTTATTTGTATAGTTCATCCATGCCATGTG-3′ introducing a *SalI* site. The amplicon was cloned into the PCR2.1 Topo plasmid, sequence was checked, the insert restricted with *PacI* and *SaII* and cloned into the pGCC2PrPilE vector restricted with the same enzymes.

### Animals

SCID/Beige (SOPF/CB17 SCID BEIGE. CB17.Cg-Prkdc-Lyst/Crl) mice (Charles River, France) 5–8 weeks of age were used (20.3±3 g). Animals were housed in disposable cages (Innovive, France) with sterile water and mouse chow.

### Grafting

Normal human skin was obtained as surgical excess from plastic surgery at Hôpital Européen Georges-Pompidou (HEGP). The superficial 200 µm of skin was harvested using a Sober dermatome (Humeca BV, Holland) and cut to approximately 2 cm^2^. Skin was stored in DMEM with 10% serum at 4°C until grafting. Grafting normally occurred within 2–4 h of surgery. Mice were anesthetized with ketamine (100 mg/kg) and xylazine (10 mg/kg) IP. Hair was removed from the posterior thorax, and a local anesthetic applied (Tronothane, Lisa Pharm). A graft bed was prepared by excising an area of skin approximately 1.5–2 cm^2^. Human skin was immediately placed over the graft bed, trimmed to size and fixed in place with Vetbond (3M, USA). Band-aids and dressing tape were applied. Dressings were removed 10–15 days post graft. Mice were infected between 21–28 days post graft. The engraftment rate was very high throughout the study with a successful graft occurring in more that 95% of cases. There was no apparent evidence of graft rejection and the very few that failed were due to physical disruption of the graft in the early stages post-surgery.

### Infection

Bacteria from overnight plates were grown to an OD_600_ 0.1 in Endo-SFM with 10% FBS (Gibco, France) at 37°C, 5% CO_2_ shaking, for 2 hours. Bacteria were washed twice in PBS and resuspended to 10^7^ CFU/ml in PBS. 100 µl (10^6^ CFU) were injected IV into anesthetized grafted mice. Mice were injected IP with 8 mg of human transferrin (Sigma Aldrich, France) prior to infection. Blood samples were taken before infection, 5 min and 6 h post infection and at sacrifice. Biopsies (4 mm) of tissues were homogenized using a MagNA Lyser (Roche, France) homogenizer. Liver, spleen and brain samples were homogenized using MagNA Lyser Green Beads (Roche, France) at 6000 rpm for 30 sec. Skin samples were homogenized using Fast-Prep lysing matrix M tubes (MPBio, France) at 6000 rpm for 2×30 sec. All homogenates were plated with dilutions immediately onto GCB agar with appropriate antibiotics.

### Sample preparation for histology and fluorescence microscopy

Tissue fixed in PFA 4% was frozen in OCT (Tissuetek, Sartorius, USA) and sliced at 10 µm. For histology, tissues were stained with hemotoxylin and eosin (H&E). For fluorescence microscopy the following reagents were used: *Ulex Europaeus Agglutinin* lectin – Rhodamine (Vector Laboratories, Eurobio ABCys, France); Monoclonal anti-human-CD31 clone JC70A (Dako, Denmark); anti-mouse CD31 (BD Pharmingen, France); Monoclonal anti-human-Collagen IV (AbD Serotec, France); anti-human-fibrinogen (Dako, Denmark); anti-CD42b clone R300 (Emfret Analytics, Germany); anti-mouse TER-119/Erythroid cells (BD Pharmingen, France); anti-mouse Ly-6G and Ly-6C, clone RB6-8C5 (BD Pharmingen, France); *Neisseria meningitidis* anti-serum Group C (Difco, USA); Polyclonal anti PilE antiserum [42]. Secondary antibodies used were: anti-Rat Alexa 488, anti-rabbit Alexa 568, (Molecular Probes Invitrogen, France) Texas-Red Streptavidin, (Vector, USA); anti-rat Cy3, (Jackson Lab USA). Monoclonal antibodies were detected using the Mouse on Mouse detection kit (Vector Laboratories, Eurobio ABCys, France). All samples were mounted in Vectashield mounting reagent (Vector Laboratories, Eurobio ABCys, France).

### Microscope set-up

Intravital and fluorescence confocal microscopy was performed using an inverted Nikon Eclipse Ti microscope equipped with a CSU-X1 M1 confocal head with a Yokogawa spinning disk head and an Evolve (Photometrics) camera (Roper Scientific, France). Images were captured using MetaMorph software (Molecular Devices, USA). Histology was imaged using a Nikon Eclipse TC600 with a Baumer camera and Archimed software (Microvision Instruments, France). Image processing was performed using MetaMorph and Image J [43]. Final figures were created in Photoshop (Adobe, USA).

### Intravital microscopy

Animals were anesthetized and a mid-line dorsal incision made from the neck to mid-back. The skin supporting the human graft was carefully separated from underlying tissue. The skin flap was attached with Vetbond (3M, USA) to a 35 mm μ-Dish with a thin bottom (Ibidi, Germany) and bathed in 37°C saline. The microscope imaging chamber was maintained at 37°C. To visualize blood flow 150 kDa-FITC dextran (TdB Consultancy, Sweden; 2 mg/animal) was injected IV. UEA lectin–Rhodamine (Vector Laboratories, Eurobio ABCys, France), was desalted, resuspended in PBS and injected IV (200 µg/ml per animal). Animals were injected IV with 10^7^ CFU of *N. meningitidis* expressing GFP.

### Cytokine measurement

Cytometric Bead Array (CBA) was performed according to the manufacturer's protocol (BD Bioscience, France). Human cytokines were detected using a combination of CBA Flex Set kits for IL-1α, IL-1β, IL6, IL-8, IL-10, IL-12p70, INFγ, INFα, TNF, MIP1α, MIP1β and GM-CSF (BD Bioscience, France).

### Statistics

Statistical data is represented as raw data points and median. Statistical analysis was performed using a two-tailed Mann-Whitney test. P values of <0.05 was considered significant. Ranking: <0.0001 = ****, 0.0001 to 0.001 = ***, 0.001 to 0.01 = **, 0.01 to 0.05 = *. Graphs were prepared using Prism (Graphpad, USA).

## Supporting Information

Figure S1 (A) A large vessel infected with *N. meningitidis* (green) at 6 h post infection. Endothelium is visualized by huCD31 labeling (red). A sloughed endothelial cell can be seen in the vessel lumen (arrow). Cell nuclei are labeled with DAPI (blue). (B) Platelet aggregation (CD42b, red) at a distance to the bacterial infection (green). (C) Platelet aggregation (CD42b, red) in the same vessel as presented in (A). (D) Fibrinogen deposition (green) in the same vessel as (A) and (C). (E) Bacterial counts in the blood (CFU/ml) and skin biopsies (CFU/mg) from mice infected with WT bacteria in the absence (− hTf) or presence (+ hTf) of human transferrin.
(TIF)

Movie S1 Intravital microscopy showing a human vessel in the base of the skin graft labeled with UEA lectin (red) and perfused by the mouse circulation. Blood plasma is labeled with a 150 kDa FITC-dextran (green) introduced intravenously. Moving black silhouettes within the plasma are blood cells.
(MOV)

Movie S2 Intravital microscopy showing adhesion of *N. meningitidis* (GFP, green) to a human vessel (UEA lectin, red) 30 min post infection.
(MOV)
