# Supplementary figures and images for: Adhesion of Neisseria meningitidis to Dermal Vessels Leads to Local Vascular Damage and Purpura in a Humanized Mouse Model
Source: PLoS Pathog. 2013 Jan 24;9(1):e1003139. doi: 10.1371/journal.ppat.1003139 (PMC3554624; doi:10.1371/journal.ppat.1003139)

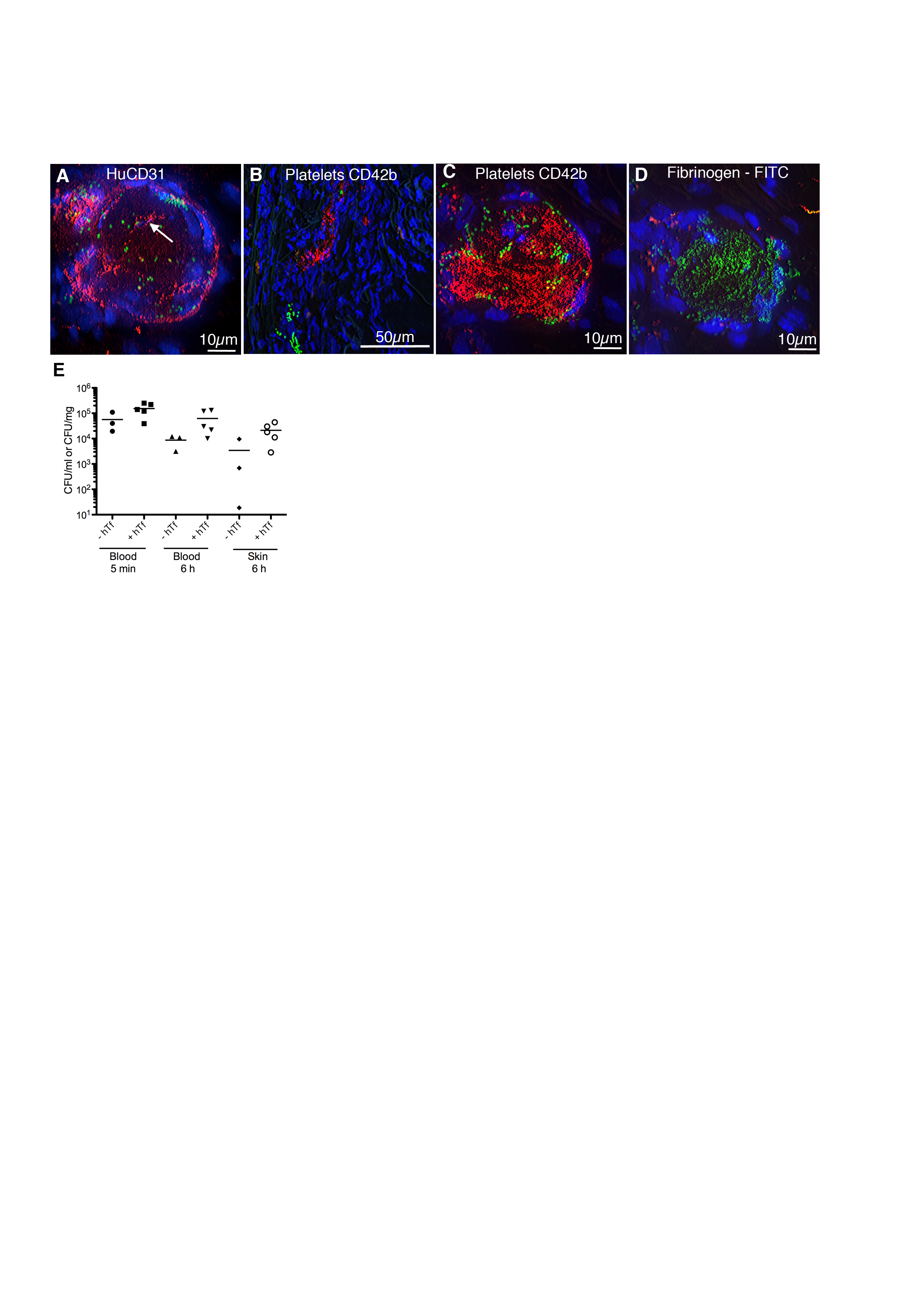

Supplement: Figure S1 — (A) A large vessel infected with N. meningitidis (green) at 6 h post infection. Endothelium is visualized by huCD31 labeling (red). A sloughed endothelial cell can be seen in the vessel lumen (arrow). Cell nuclei are labeled with DAPI (blue). (B) Platelet aggregation (CD42b, red) at a distance to the bacterial infection (green). (C) Platelet aggregation (CD42b, red) in the same vessel as presented in (A). (D) Fibrinogen deposition (green) in the same vessel as (A) and (C). (E) Bacterial counts in the blood (CFU/ml) and skin biopsies (CFU/mg) from mice infected with WT bacteria in the absence (− hTf) or presence (+ hTf) of human transferrin. (TIF) [file ppat.1003139.s001.tif]
